# Using Visual and Digital Imagery to Quantify Horn Fly (Diptera: Muscidae) Densities

**DOI:** 10.1093/jisesa/ieaa110

**Published:** 2020-11-02

**Authors:** Brandon Smythe, David Boxler, Gary Brewer, Eric Psota, D Wes Watson

**Affiliations:** 1 The Center for Animal Health and Food Safety, New Mexico State University, Las Cruces, NM; 2 Department of Entomology, University of Nebraska, Lincoln, NE; 3 Department of Electrical & Computer Engineering, University of Nebraska, Lincoln, NE; 4 Department of Entomology & Plant Pathology, North Carolina State University, Raleigh, NC

**Keywords:** veterinary entomology, pest management, surveillance

## Abstract

The horn fly, *Haematobia irritans* L. (Diptera: Muscidae), is a persistent pest of cattle globally. A threshold of 200 flies per animal is considered the standard management goal; however, determining when that threshold has been exceeded is difficult using visual estimates that tend to overestimate the actual fly densities and are, at best, subjective. As a result, a more reliable and durable method of determining horn fly densities is needed. Here, we describe the methods commonly used to quantify horn fly densities including visual estimates and digital photography, and provide examples of quantification software and the prospect for computer automation methods.

The horn fly, *Haematobia irritans* L., is a persistent and important pest of pastured cattle regardless of the production system (Drummond 1987, Byford et al. 1992, Jones 2002, Nickerson 2016). As an obligate blood-feeding parasite of cattle, the horn fly takes multiple bloodmeals per day (Harris et al. 1974) inducing stress, altering grazing habits, and decreasing milk production and weight gains (Harvey and Brethour 1979, Harvey and Launchbaugh 1982, Boland et al. 2008, Mays et al. 2014, Mullens et al. 2017). In fact, Georgia beef producers estimated that horn fly infestations of pastured cattle result in economic losses exceeding $5.9 million per year in that state alone (Hinkle 2018). Furthermore, horn fly associated losses throughout the United States were estimated to be between $700 million to $1 billion per year (Nickerson 2016). The impacts associated with horn fly infestations are not limited to animal performance as evidence of hide damage attributed to horn fly feeding likely reduces leather quality (Guglielmone et al. 1999). Moreover, horn flies have been incriminated in the transmission of *Staphlococcus aureus*, the bacterium commonly associated with bovine mastitis (Edwards et al. 2000, Oliver et al. 2005, Anderson et al. 2012) and can serve as the intermediate host for *Stephanofilaria stilesi*, a nematode causing granular dermatitis (Hibler 1966, Dies and Pritchard 1985).

Horn flies require fresh, undisturbed cattle dung to complete immature development, which can occur in 9–12 d under ideal weather conditions. Furthermore, horn flies will diapause as pharate adults within the puparium beneath dung pats during the cooler winter months and emerge as adults the following spring (Hoelscher et al. 1967, Thomas 1985). In response to environmental influences, patterns and timing of adult population emergence, growth, and characteristic seasonal peaks fluctuate regionally. As such, monitoring and estimating adult horn fly populations associated with individual animal herds has become the key first step in employing effective Integrated Pest Management (IPM) programs.

Management of horn flies is typically initiated by monitoring population densities to determine the need for timely interventions and treatments. Historically economic thresholds have fallen in a range between 50–230 flies per animal (Haufe 1979, Butler and Okine 1999, respectively). Today the generally accepted standard is 200 flies per animal (Schreiber et al. 1987, Hogsette et al. 1991). Inherent to the economic threshold is the need to accurately estimate horn fly populations, which have historically been based on visual counting techniques.

Determining the density of horn flies on an animal can be challenging. In rare cases, estimates of 10,000 flies on a single animal have been reported in untreated herds (Bruce 1964). In addition to high populations, horn flies take flight and temporarily leave the host in response to defensive animal behaviors. At any moment, a cloud of flies can flush (rise from the back of the host) and land again on the same host within seconds. Flies may also flush from one side of an animal only to land on the opposite side or on another animal.

The propensity of horn flies to flush and the large variation in numbers from region to region make standardizing a visual counting methodology problematic. While both side counts remain the most common visual count method (Morgan 1964, Lysyk 2000), they can be difficult to complete, particularly when large numbers of flies are present and before flies flush, and disrupt the count. An alternative is to take visual counts from one side of the host animal and double the number to estimate full populations. But this technique also has its faults, especially if the sample size is small, as flies are not distributed equally from side to side on the host.

As researchers practice visual estimation of horn fly densities, accuracy improves. However, such estimates are viewed as subjective and tend to vary among observers. Still images or recordings of infested animals have been used to overcome potential problems with visual estimations of horn fly densities. Prior to the late 1990s, enumerating horn flies on cattle in pasture settings required binoculars, particularly if cattle were unaccustomed to human contact (Tugwell et al. 1969; Williams and Westby 1980, Skoda et al. 1987). Using a 35-mm single-reflex lens camera equipped with a telephoto lens was an option but it was expensive and introduced a time delay in processing the film into a print or 35-mm slide. Counting horn flies from a print also presented significant challenges, as limitations in the resolution and dynamic range of prints limit the observer’s ability to discern individual flies. Projecting the image onto a screen increased counting accuracy, but the process was cumbersome and prone to subjective error.

A number of investigations have been conducted to evaluate the effectiveness between and within multiple horn fly counting techniques. For instance, Lima et al. (2002) compared visual population estimates taken by a trained observer to video recordings taken from the back of a horse to allow for a close interaction with the cattle. Results indicated that trained observers underestimated horn fly densities relative to the recorded data. In contrast, Castro et al. (2005) conducted a study to validate the horn fly counts of a trained observer by comparing video recordings taken moments after the visual count. Animals were placed in a chute one at a time and two observers simultaneously counted flies on the backs of the animals from above. The comparative recordings were made immediately following the visual count and closely approximated the observers counts (*r*^2^ = 0.98). However, accuracy of the visual estimates decreased with higher densities of flies on animals, further supporting complications when utilizing visual estimations (Castro et al. 2005).

The availability of high-quality digital cameras to capture images of a horn fly infested animal has been suggested to be able to improve the practicality of estimating counts from images. Still images can be taken from one or both sides of the animal along with the numbered ear tag for accurate animal identification (Pruett et al. 2003, Untalan et al. 2006, Boland et al. 2008, Mochi et al. 2009, Mullens et al. 2016). Unfortunately, this counting method, like visual estimations, is not without challenges. Cattle avoidance behaviors to human interaction can make it difficult to obtain a clear image of both the animal and the flies. As a result, multiple images may be required to achieve a clear image that can be used for accurate estimation. In addition, focal points of the captured image may omit certain areas of the body, making estimations in areas of the head, legs, and underside of the belly difficult if not impossible (Mullens et al. 2016). Quantification of the flies in the images is also challenging if projected onto a large screen and then counted manually. This is particularly difficult if the fly densities are too numerous to count.

For purposes of the current document, the authors suggest that researchers concerned with estimating horn fly population densities utilize either visual or still image estimation techniques. The techniques described below are offered as a generalized guide, as these techniques—particularly those associated with still image capturing—are likely to improve in the future.

## Procedures

When initiating a project that will assess horn fly populations, researchers will first need to identify the counting method that best meets their research objectives and local conditions. It is highly recommended that studies adhere to either visual or image-based estimations and avoid mixing these techniques within a single project to ensure consistency across time. In addition, research objectives and study environments will provide insight as to the most appropriate method.

**Fig. 1.**
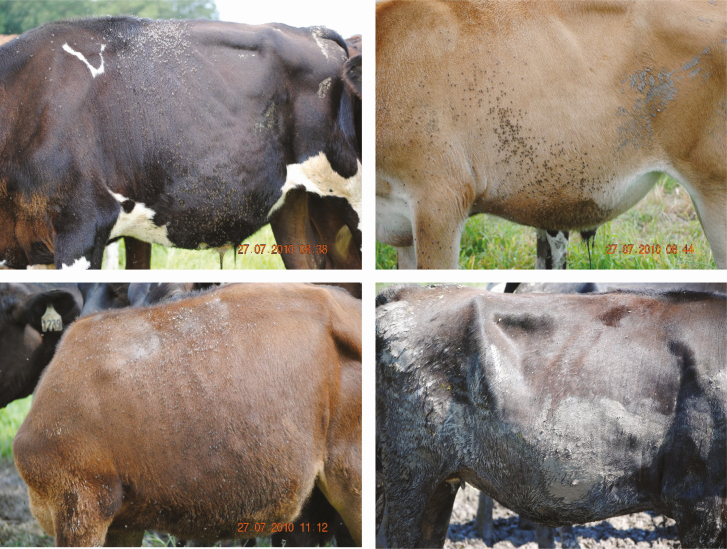
Accurate determination of horn fly densities can be influenced by animal color, presence of dirt or mud, and lighting aspect. Images by D. W. Watson.

When conducting horn fly surveillance, it is important to make observations consistent with the project protocol, i.e., on the same day and time of day, weather permitting. When conducting observations, it is good practice to document the temperature and weather conditions, e.g., clear or cloudy skies. Horn fly observations are best conducted in the morning between the hours of 0800 and 1100 a.m. when horn flies are on the back and sides of an animal and less likely to be on the lower body regions (Schreiber and Campbell 1986). Flies can be readily seen on light or dark colored animals but are less obvious on brown or dirty animals (Fig. 1).

## Visual Estimations

Visual horn fly population estimates provide researchers with the ability to rapidly collect data and bypass the need for post collection processing associated with digital photographs. However, in alignment with the limitations discussed previously, researchers should be aware of the potential problems associated with these techniques. Regardless, visual population estimates remain a valid and reliable technique for measuring horn fly population densities. The following suggestions are based on the authors experience in this field and are presented as a generalized recommendation to visually assess horn fly populations.

The accuracy of visual observations is difficult or impossible to confirm as estimates vary based on experience (Smythe et al. 2017). As such, consistency within research projects should be pursued. Observations should be taken by the same person throughout the duration of the project to maximize consistency in population estimations. Often, researchers estimate horn fly populations for a herd by sampling a subset of individual animals (Krafsur and Ernst 1986; Schreiber and Campbell 1986). If using subsets of animals to estimate herd averages, efforts should be made to evaluate the same animals at each data collection event, as individual animals within a herd may vary in attractiveness (Franks et al. 1964; Pruett et al. 2003). Typically, population estimates within a season are evaluated on a weekly basis. However, sampling frequencies may change depending on study objectives. For example, when evaluating repellent products on horn fly populations, daily or even hourly population estimates are often utilized (Lachance and Grange 2014, Mullens et al. 2017).

Although binoculars have been used in previous studies (Tugwell et al. 1969, Williams and Westby 1980) to estimate horn fly populations, visual counts taken from approximately 1–3 m away have also been used (Schreiber and Campbell 1986, Smythe et al. 2017) and are suggested when cattle are easily approachable. Researchers may benefit from the use of a hand-held tally counter to help with the counting procedures. Beginning at the head of the animal and moving towards the tail, researchers should count one side of the animal taking special care to account for flies on the backline and underbelly prior to moving to the opposite side and repeating these procedures to capture full body estimates. When fly populations are high, it may benefit researchers to count groups of flies (i.e., count by 5, 15, or 25). It should be noted that fly and animal movement are likely to influence population estimates. Alterations to the suggestions presented here are likely to occur due to the nature and scope of individual projects.

## Digital Estimations

High-quality digital cameras provide clear images and are typically within the range of 14–24 megapixels: Nikon 5300 (Nikon Corporation, Minato-ku, Tokyo, Japan) and Canon SX510 HS (Canon Inc., Lake Success, NY) (Smythe et al. 2017).

**Fig. 2.**
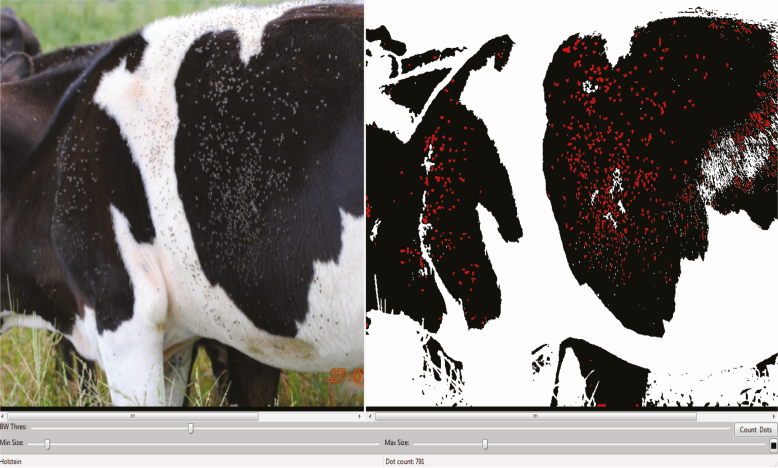
MIT software DotCount (http://reuter.mit.edu/software/dotcount/) was used to estimate the number of horn flies on one side of a dairy cow. The human estimate was 1,100 flies, the DotCount estimate was 791 in black areas (shown on right) and 181 in white areas (not shown) for a total of 972 flies, and the HDTV grid count was 1,053 horn flies. Cow image by D. W. Watson, DotCount template by http://reuter.mit.edu/software/dotcount/.

When taking a digital image, the colors of the animal can influence the ability to detect flies either visually or with counting software. For example, estimations of a fly load from a digital photograph of the side of a black and white cow uploaded into the online MIT DotCount system (http://reuter.mit.edu/software/dotcount/) were compared to the estimate of a trained observer viewing the same image. The software uses contrast to generate a count of the desired objects (Fig. 2) and the trained human observer uses experience to estimate the number of flies. Lastly, the image was visualized on a 236.22-cm high-definition television (HDTV) with a gridwork overlay to partition the image into subsections and the individual flies were counted and recorded within each subsection. Results from the visual estimate performed by the trained observer were higher than the HDTV count and the DotCount method. The higher visual estimate confirms that humans tend to overestimate the fly densities (Mullens et al. 2016, Smythe et al. 2017) relative to digital-based assessments.

**Fig. 3.**
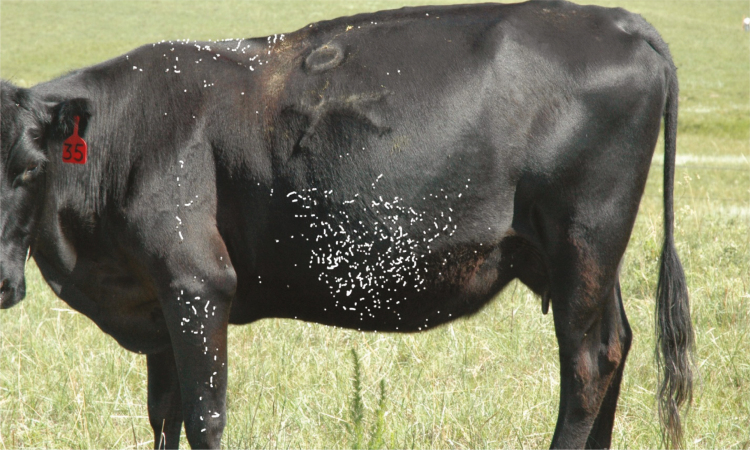
Horn flies identified and enumerated with GIMP 2.10.18, GNU Image Manipulation Program. Image by D. Boxler.

Digital imagery technology for enumerating horn fly numbers has been used at the University of Nebraska, West Central Research & Extension Center since 2008 (Boxler et al. 2018). The protocol for horn fly photography requires a digital camera with a minimum of 24-megapixels and a 28- to 300-mm lens. Digital imagery of horn flies is conducted between the hours of 0800 and 1100 a.m. when horn flies are typically found on the top line and sides of cattle (Schreiber and Campbell 1986). Because cattle dispersed in the large pastures typical of semi-dry environments are cautious of approaching people, horn fly assessments are recorded from one side of 15 randomly selected animals from a herd. By collecting images from single sides of 15 cattle, the side-to-side variation of fly distributions (as noted earlier in this paper) is assumed to be even. Counts are typically made on a weekly schedule through the fly season. Recorded images are viewed using the computer imaging program GIMP 2.10.18 (GNU Image Manipulation Program). The count for each of the 15 images are doubled to estimate the total number of horn flies per animal (Fig. 3).

Fly count data may be analyzed using a variety of statistical methods suitable to determine treatment effects. These methods often involve data transformation, repeated measures, least square means, and weekly percent reduction relative to the untreated control.

## Future Prospects

Deep learning and computer vision provide a promising path forward toward automating fly counts from digital images of cattle. Traditional image processing methods that rely on techniques like pixel intensity thresholding and connected components are prone to failure in the presence of textured fur, mud splatters, and specular reflections. In contrast, deep learning allows the computer to handle all manner of presentations by learning directly from human annotations. These algorithms learn to recognize flies much like a human, where features from both the flies and the background are identified and synthesized before deciding whether a given part of the image contains a fly.

**Fig. 4.**
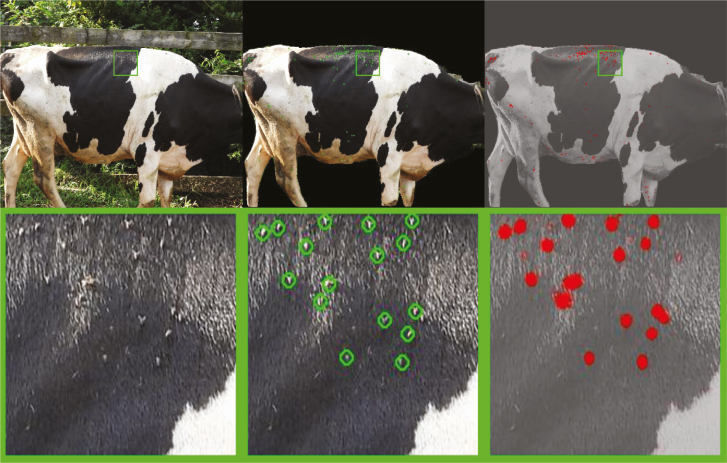
Deep learning output for fly counting. The top row shows the original image view and the second row focuses in on the crop defined by the green rectangle. The left image is original capture, the middle image shows the detections output by the deep learning detector, and the right side illustrates the raw network output where red regions are where the network thinks flies are located on the cow. Cow images by G. Pigetti and E. Luc, UT.

Common object detector frameworks like YOLO (Redmon et al., 2016) and DeepLabV3+ (Chen et al. 2018) can easily be adapted to tasks like fly counting to achieve impressive results. After using transfer learning to train DeepLabV3+ with a pretrained ResNet18 backbone (He et al. 2016) on 414 human-annotated images, this network demonstrates the ability to detect individual flies in both dark and light regions on a black and white cow (Fig. 4). The zoomed-in crop shows the relatively low level of detail that the network uses to identify flies, where each fly could fit within a 10 × 10 pixel window. Still, even in the presence of unwanted reflections on black fur, the network correctly identifies each of the flies in this region.

**Fig. 5.**
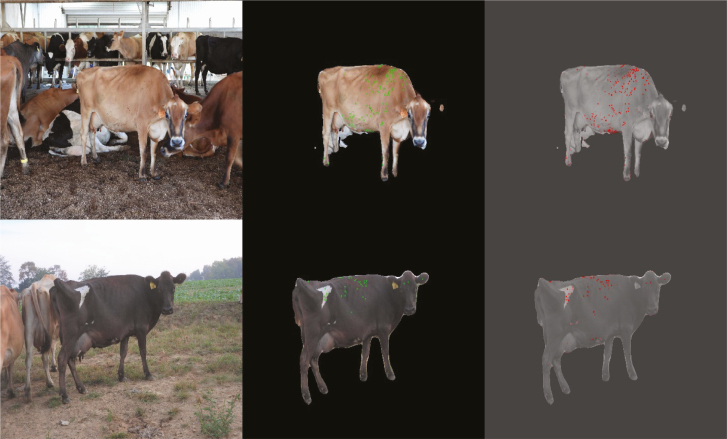
Salient cow segmentation used to isolate the cow of interest from all other cows in the image. The salient cow is defined as the cow that overlaps with the center pixel of the image. Cow images by G. Pigetti and E. Luc, UT.

Another consideration that must be made while developing automated approaches is the presence of unwanted cattle in the field of view. Whereas the person capturing a picture knows which cow in a scene is being targeted for a fly count, the computer does not. Until recently, segmenting and separating each individual cow in a crowded scene would be an insurmountable task for computer vision. However, deep learning networks designed for pixel-wise segmentation make it possible to isolate areas of the image corresponding to the cow of interest. The results (Fig. 5) were generated after training a network based on the DeepLabV3+ architecture to isolate the cow in the center of the image from all other cows. Note that without using segmentation in situations where multiple cows exist in the image, flies on these neighboring cows could easily double or triple the fly count that would be obtained.

## Summary

We have provided background on horn fly surveillance methods put to practice and discussed the pitfalls, how digital imagery can improve archival data collection, and how future technology using machine learning can improve the efficacy of data collection from digital images. Regardless of the horn fly surveillance method used, it is good practice to be consistent when making observations with the day of the week, time of day and document the weather conditions. Conduct observations in the morning between the hours of 0800 and 1100 when it is cooler and flies tend to be on the upper body regions. Observations can be taken from one or both sides of the animal depending on local conditions. Visual assessments of horn fly densities tend to overestimate the actual number and, while digital photography is less subjective and provides a durable image source for reference, it requires an extra counting step. Looking forward, as deep learning and computer vision software continue to advance, the automated quantification of flies from digital images is expected to become routine as a viable tool for researchers and producers.
